# Transdisciplinary education for planetary health

**DOI:** 10.3389/fmed.2025.1731290

**Published:** 2026-02-19

**Authors:** Teddie Potter

**Affiliations:** University of Minnesota Twin Cities School of Nursing, Minneapolis, MN, United States

**Keywords:** climate change, interprofessional, planetary health, systems thinking, transdisciplinary

## Abstract

The level of ecosystem integrity that is essential for life on the planet is threatened, and the changes we are witnessing indicate that we are close to irreversible tipping points. Planetary crises such as climate change, biodiversity loss, and pollution are impacting human health and threatening previous gains in global health. These are not future risks; they are current events, and we must prepare health professionals to provide competent care in a system that is not prepared for a climate-changed world. Health professionals must be educated about the nature of these threats, the cause of the crises, methods to identify and prevent new and emerging health impacts, and strategies to advocate for mitigation even while ensuring that patients, families, and communities are prepared to adapt to the changes. This perspective piece calls on health profession educators to move curriculum content beyond climate change to the full scope of planetary health and to pivot from interprofessional education toward transdisciplinary education and systems thinking.

## Introduction

A recent statement captures the current reality: “It’s not just climate change; it is everything changing.” We are witnessing massive upheaval across social and ecological systems worldwide, and these changes are threatening human health. The impacts are so significant and happening with such frequency and intensity that our health professionals and healthcare systems are not adequately prepared. Consequently, health professional education must incorporate new content and new pedagogy.

Health professionals recognize that it is challenging for patients to survive multi-system failure, and if interventions cannot restore balance, death ensues. The same is true for the earth. According to the Stockholm Resilience Center, there are nine interconnected systems—or *planetary boundaries*—that must not be exceeded if society, and life as we know it, are to be supported. These boundaries include climate change, biodiversity loss, land system change, freshwater change, modification of phosphorus and nitrogen flows, ocean acidification, stratospheric ozone depletion, increase in atmospheric aerosol loading (air pollution), and introduction of novel entities (chemical pollution) (1). The *Planetary Health Check 2025* reports that, in 2025, we exceeded seven of the nine boundaries that are necessary for supporting life on the planet (2). The planetary crises that we currently face are significantly impacting human health globally; however, health professionals have not been educated to prepare for or adapt to this urgent crisis. We need to significantly transform health profession education and continuing education of health professionals to save lives and promote wellbeing.

## Background

### Curriculum content

#### Climate and health education

Although the health impacts of climate change were discussed as early as 1989 (3), the majority of the climate discussions focused on sustainability, as health systems began to realize that energy consumption and the costs associated with supply procurement and waste management were affecting the bottom line. At that time, climate mitigation was not the focus of efforts; business was.

Climate change and its impact on health began to appear in health profession education in 1997 when Harvard Medical School offered a course on human health and global environmental change (4). Columbia University’s Global Consortium on Climate and Health Education (GCCHE), founded in 2017, provided some of the earliest examples of climate and health content for education (5), about. Further support emerged in 2016 with the establishment of the Medical Society Consortium on Climate and Health (MSCCH), which represents more than 70% of American physicians in 38 national societies and other health profession organizations. It educates its members on emerging issues related to climate and health and how to advocate for policy changes to protect health (6).

In 2017, the University of Minnesota published one of the first comprehensive interprofessional climate curricula for health professions (7). The initiative began with a survey of over 200 health profession students who indicated that they would be practicing in an era in which patients present with climate-related issues and that their university programs were not preparing them to practice competently in relation to climate change. The student survey results were presented to the deans of all health science programs, including nursing, medicine, pharmacy, public health, dentistry, veterinary medicine, and allied health. Upon hearing the concerns of the students, the deans recommended that climate content be added to the curriculum of every school.

An interprofessional team composed of representatives from every health profession school came together to create an interprofessional slide deck that includes topics such as an overview of climate and health, air quality, heat, extreme weather, vector changes, and other impacts. Key values of the project are that the slide decks are to be easy to use by faculty, that the slide decks are applicable to be used by any health discipline, and that the decks are open access for the university and beyond (8).

Lemery et al. (9) identified a lack of guides and resources to allow for the evaluation of learners’ climate knowledge and practice competencies. In response, they proposed a framework to structure progressive learning of climate content across shared curricular learning domains for pharmacists, psychologists, social workers, nurses, and physicians. This framework represents a significant addition to the literature and can be easily adopted to incorporate planetary health themes, specific topics, and measurable competencies.

#### Planetary health education

**Figure 1:**
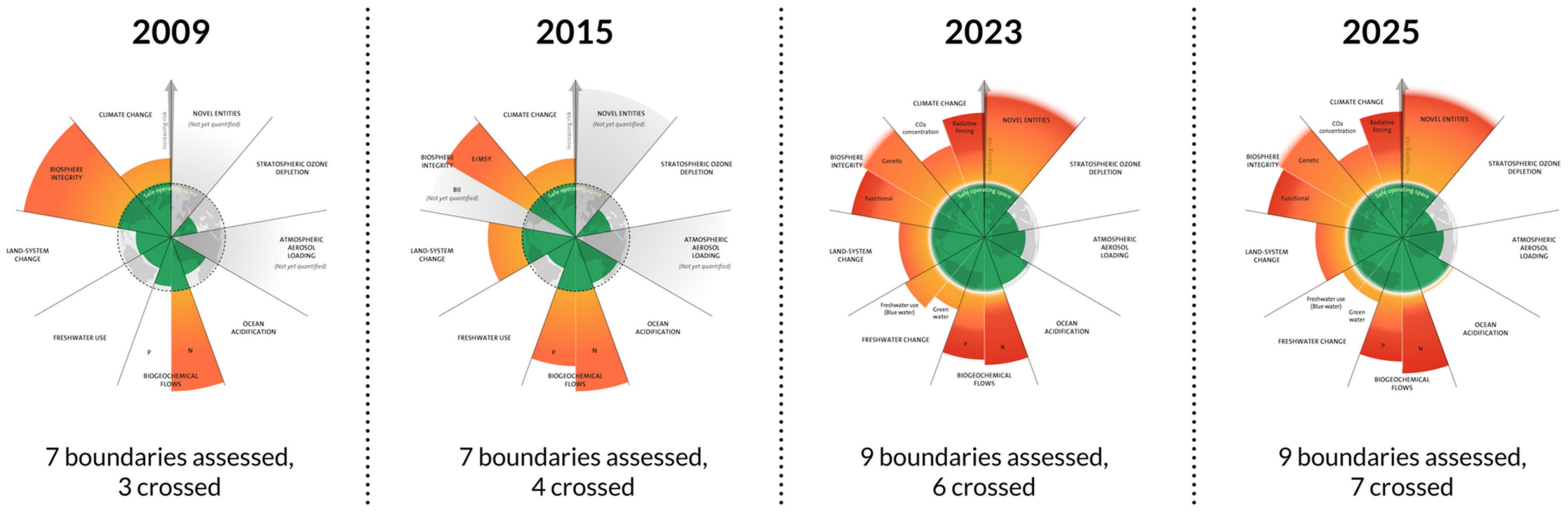
Evolution of the planetary boundaries’ framework. Reproduced from “Planetary boundaries” by Stockholm Resilience Centre (https://www.stockholmresilience.org/), licensed under CC BY-NC-ND 3.0 (Credit: Azote for Stockholm Resilience Centre, Stockholm University). Based on the studies by Richardson et al. (27), Steffen et al. (28), and Rockström et al. (29).

The United Nations recognizes that the environmental crisis is not limited to climate change. Urgent threats also include biodiversity loss and pollution; therefore, the UN recognizes this as the “Triple Planetary Crisis” (10). Research conducted by scientists at the Stockholm Resilience Center (1) has found that there are 9 interconnected systems that work together to support life on the planet. Threats to these systems include changes in biosphere integrity, land system change, freshwater change, modification of biogeochemical flows (phosphorus and nitrogen), ocean acidification, increase in atmospheric aerosol loading (air pollution), stratospheric ozone depletion, introduction of novel entities (chemical pollution), and climate change (Figure 1).

These nine *planetary boundaries* make up the earth’s life support system and must not be exceeded; however, in 2025, a seventh boundary (ocean acidification) was breached (11).

One can see that climate change is only one of many environmental threats to human health. Therefore, this article suggests that health profession education move beyond climate change toward planetary health, as planetary health analyzes and addresses the full range of human-caused environmental issues.

**Figure 2:**
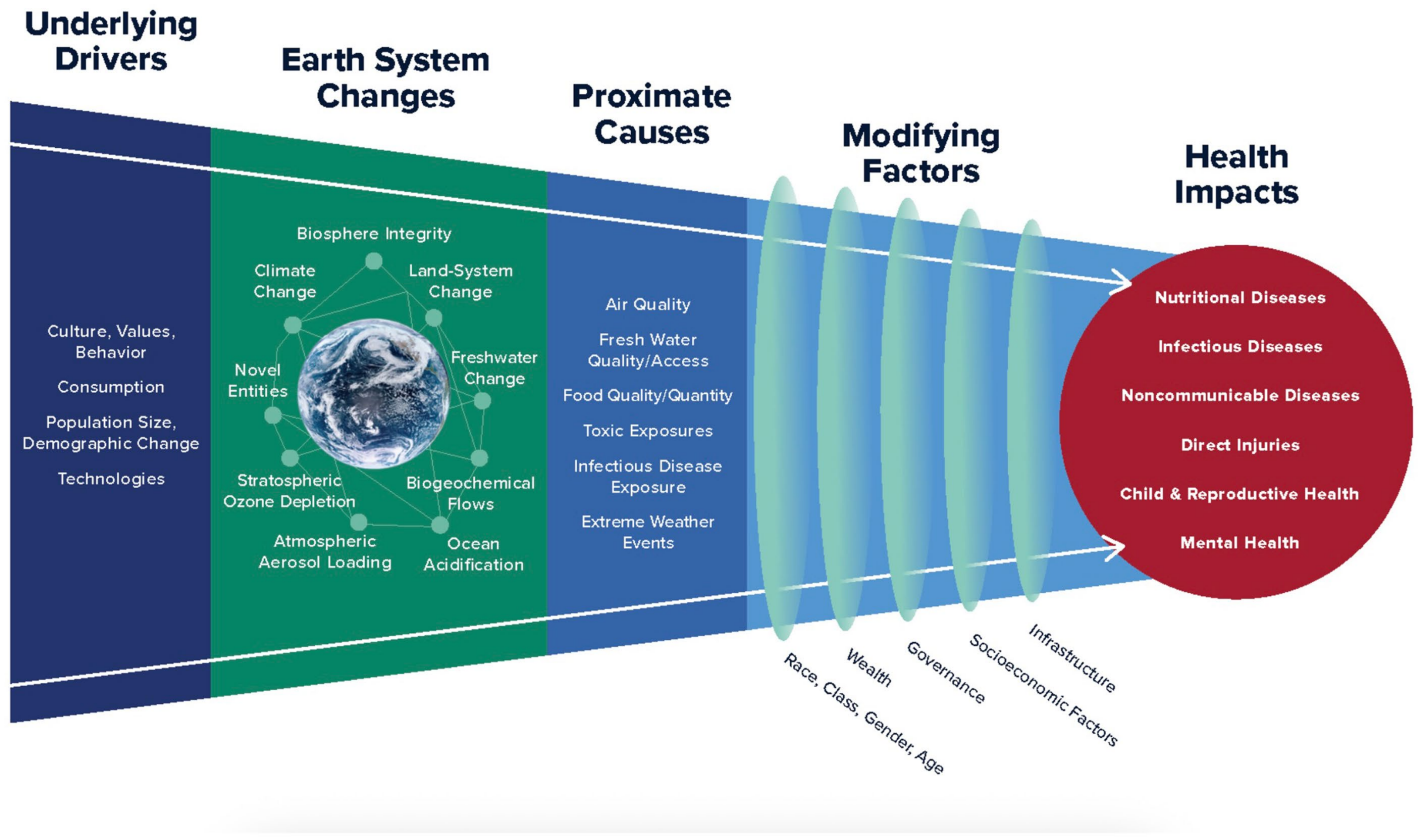
Planetary health: protecting human health on a rapidly changing planet. Reproduced with permission from “Planetary Boundaries Schematic” (https://planetaryhealthalliance.org/planetary-health-schematic/) by Planetary Health Alliance’s (PHA).

Planetary health is defined as “a solutions-oriented, transdisciplinary field and social movement focused on analyzing and addressing the impacts of human disruptions to Earth’s natural systems on human health and all life on Earth” (12). Figure 2 illustrates the interconnection of the root causes of these problems, the earth system or planetary boundaries, the ecosystem changes, and the resulting health impacts. The figure also illustrates that impacts are not equitable. Social determinants of health, including ethnicity, socioeconomic factors, and structural determinants such as government policies, cause planetary impacts to be experienced differently and recovery and adaptation to be extremely complex.

Future health professionals must be educated to think in terms of systems and complexity. Learning about climate change is not sufficient. To be effective, they will need to grasp the multiple levels of environmental degradation and the human behaviors that need to be addressed to prevent the worst consequences predicted for the future.

### Pedagogy

Once the decision is made to include climate or planetary health content in the curriculum, it is equally important to select a pedagogical approach that fosters “transformative, inclusive, integrative—and sometimes disruptive—approaches to learning that strengthen our capacity to work toward the goals and imperatives of planetary health” (13), p. e77.

#### Interprofessional pedagogy

As noted, since its earliest days, climate change education has been interprofessional, largely because the impacts of climate change cut across sectors. Moreover, no one discipline can address the complex impacts alone. “Interprofessional education occurs when students from two or more professions learn about, from, and with each other to enable effective collaboration and improve health outcomes” (14). The advantages of interprofessional education are that it creates a sense of teamwork and collaboration that are necessary for health or, in the case of climate change, strengthening mitigation and adaptation efforts. It also promotes an awareness of disciplinary strengths that allow teams to function effectively and efficiently. For example, interprofessional education helps students understand that physicians are skilled at diagnosing and treating emerging diseases, whereas nurses are skilled at teaching patients and families about disease and symptom management and lifestyle modifications to adapt to changes.

#### Transdisciplinary pedagogy

Planetary health is vigorously transdisciplinary. In addition to being interprofessional and inclusive of diverse professions, the planetary health movement includes people from all walks of life and all levels of education, not only professions or college graduates. Planetary health is also mindful of *how* people come together and not simply that they do come together.

Transdisciplinarity can be described as creating a deep and respectful environment where an exchange of ideas can occur and new insights and initiatives become possible (15). Transdisciplinarity is the relational context for tomorrow’s solutions. It also disrupts professional hierarchies that stand in the way of innovation.

One approach to creating a transdisciplinary mindset is to have students read and analyze core documents from another discipline. For example, medical students could read the American Nurses Association (16) *Code of Ethics for Nurses*. Nursing students could be asked to read the *Declaration of Alma-Ata* (17), a global agreement on the important role that primary healthcare plays in advancing global health. Both groups of students could then be asked to describe one new structural change for the American healthcare system based on insights from both documents.

Students can also be encouraged to engage with disciplines beyond traditional healthcare; for example, helping students connect health to agriculture, engineering, and the arts. This level of transdisciplinary interaction promotes systems thinking, a greater understanding of complexity, and a deeper awareness that health must be the foundation for all human endeavors.

Putting this all together, it is recommended that we expand beyond climate change and interprofessional education to planetary health transdisciplinary education. Only by doing so will we address the complexity and urgency of the current crisis.

#### Core resources for transdisciplinary education for planetary health

If we are to create transdisciplinary relations that foster collaboration between disciplines as different as nursing, farming, and engineering, then it is important to have a shared language, shared vision, and shared strategy. The Planetary Health Alliance provides exceptional resources for faculty in all fields (18).

### *Planetary Health Education Framework*: our shared language

The *Planetary Health Education Framework* (19) describes the five core domains that provide students with a planetary lens for their lives and practice. The core domains offer a shared language to support collaborative work and understanding. These five core domains include the following:

- *Interconnection within nature:* Humans are not separate from nature but rather embedded in nature. What you do to the rest of nature, you do to yourself. Human health relies on the health of the entire ecosystem. As trusted voices, health profession students can be educated to draft and support policies that support ecosystem health.
- *The Anthropocene and health:* The threats to planetary health are not natural; they are human-caused; therefore, human behaviors need to change. Health profession students can be instructed to address structural and social determinants of health as part of their professional obligations. They can teach their patients about the health impacts of environmental destruction and how to change behaviors in their own lives.
- *Equity and social justice:* As Figure 1 illustrates, the health impacts of the planetary health crisis are felt disproportionately, with those who have contributed the least to the problem frequently suffering the most. It is essential for health professionals to understand these impacts and advocate for health equity in practice, research, and policies.
- *Systems thinking and complexity:* Western medicine has a history of defining health and the role of health professionals too narrowly. Students must be educated to recognize health in all things and in all places. They must grasp the complexity of the entire system to avoid creating interventions that help one part of the system while creating unintended consequences for other parts of the system.
- *Movement building and system change:* Movements and change do not happen spontaneously. The most effective movements are evidence-based, strategic, and built on collaboration between various stakeholder groups. Health profession students need to understand that change does not rest on their efforts alone. They are invited to be part of a much larger network that advocates for and works toward lasting structural changes.

### *São Paulo Declaration on Planetary Health*: our shared vision

It is not enough to be able to effectively communicate with one another; we also need a shared vision for the future. The São Paulo Declaration *on Planetary Health* (20) is a global crowd-sourced document that offers a blueprint for a Great Transition, or massive redesign, of all institutions—from research and education to manufacturing, urban planning, and economics.

### *Planetary Health Roadmap and Action Plan:* our shared strategy

Three areas need special attention if structural determinants of planetary health are to be adequately transformed at the scale and urgency required: governance, business, and education. The *Planetary Health Roadmap and Action Plan* (21) proposes transdisciplinary strategies to transform these sectors globally. This work remains under development and continues to evolve as new technologies, such as artificial intelligence (AI), and global threats and challenges arise.

### Students call for transdisciplinary education for planetary health

As educators of health professionals, our greatest commitment should be to the learning needs of our students. Two significant statements reflect the student voice. In May 2025, the International Council of Nurses (ICN) published a topic brief titled *Nursing for Planetary Health and Well-Being* (22). The brief concludes, “By adopting a planetary health lens, embracing transdisciplinary collaboration, and centering equity and Indigenous knowledge, nurses can help shape a healthier, more just and sustainable future for all life on Earth” (p.8). In response to the brief, the ICN Nursing Student Steering Group and the ICN Alliance of Student and Early Career Nurses drafted a statement of support. They wrote, “We stand united in our deep concern for the health of our planet and the profound implication this has for health and the future of nursing practice. As future professionals and emerging leaders, we carry the responsibility to be part of the solution” (23), p. 1.

Another resource for student feedback and participation is the *Planetary Health Report Card*, a global student-led movement in which health profession students grade their own institutions on how effectively planetary health is included in their curriculum, research, community outreach, and advocacy. Currently, there are Planetary Health Report Cards (PHRC) for medical schools, nursing, pharmacy, dentistry, physiotherapy, occupational therapy, veterinary, nutrition and dietetic sciences, healthcare management, and audiology (24), about us. It is noteworthy that the Planetary Health Report Card has become a rapidly growing global movement that included 188 health professional schools from 21 countries and 10 disciplines in its 2025 report (25). The report acknowledges inadequate representation from Africa, Asia, and South America, and targeted outreach has been set as a priority for 2026.

For faculty who want to include planetary health content in their curriculum, the Planetary Health Alliance offers a “curated collection of resources designed to empower educators in inspiring the next generation of leaders and advocates” (26). The resources are fully open access and therefore available to people from low- and middle-income nations.

Our future health professionals have spoken. They believe they need planetary health education to practice effectively and without negligence. In the words of the student nurses, “Because there is no health without a healthy planet and there is no future of health without the education of nurses who are prepared to protect it. Nurse the planet. Heal the future” (23), p. 2.

## Discussion

Human-caused disruptions of the Earth’s natural systems come with significant impacts on local and global health. To address these challenges with the necessary scale and urgency, thoughtful strategies and cross-sector collaboration are required. Old structures of education must be modified to include planetary health content and transdisciplinary relationships.

The schools and universities that move in this direction will likely attract the most talented students and faculty and lead the development of innovative solutions. All health professions and health profession education schools are invited to join the global movement offered through the Planetary Health Alliance, since health is only possible when it includes the planet.

## Data Availability

The original contributions presented in the study are included in the article and further inquiries can be directed to the corresponding author.

## References

[1] Stockholm Resilience Center. (n.d.). Planetary boundaries. Available online at: https://www.stockholmresilience.org/research/planetary-boundaries.html (Accessed October 17, 2025).

[2] Kitzmann N.H. Caesar L. Sakschewski B. Rockström J. (2025). Planetary boundaries science: planetary health check 2025. Available online at: https://planetaryhealthcheck.org (accessed October 22, 2025).

[3] Leaf A. Potential health effects of global climatic and environmental changes. N Engl J Med. (1989) 321:1577–83. doi: 10.1056/NEJM198912073212305, 2511446

[4] Marill MC. Pressured by students, medical schools grapple with climate change. Health Aff. (2020) 39:2050–5. doi: 10.1377/hlthaff.2020.01948, 33284698

[5] Columbia University Global Consortium on Climate and Health Education. (n.d.). About. Available online at: https://www.publichealth.columbia.edu/research/programs/global-consortium-climate-health-education/about (Accessed October 22, 2025)

[6] Sarfaty M Duritz N Gould R Mitchell M Patel L Paulson J . Organizing to advance equitable climate and health solutions: the medical society consortium on climate and health. J Climate Change Health. (2022) 7:100174. doi: 10.1016/j.joclim.2022.100174

[7] Demorest S. Potter T. (2019). Chapter 7: Climate change and health: an interdisciplinary exemplar. In: Kremers K. L. Liepens A. S. York A. M. (Eds.), Developing change agents: Innovative practices for sustainability leadership. Minneapolis, MN USA: University of Minnesota Libraries. Available online at: https://open.lib.umn.edu/changeagents/ (accessed October 22, 2025).

[8] University of Minnesota Academic Health Center Office of Education. (2018). Climate change and health: an interprofessional response. Available online at: https://globalhealthcenter.umn.edu/climate-change-and-health-curriculum (Accessed October 22, 2025)

[9] Lemery J O’Connor T Gillespie E Blackburn H Demorest S Philipsborn R . Opportunities for strengthening climate education for clinical health professionals. NAM perspectives 2024. NAM Perspectives 2024. (2024) 1–11. doi: 10.31478/202409b (accessed October 22, 2025). PMC11784527 39896746

[10] United Nations. (2022). What is the triple planetary crisis? Available online at: https://unfccc.int/news/what-is-the-triple-planetary-crisis (Accessed October 22, 2025).

[11] Potsdam Institute for Climate Impact Research. (2025). Planetary Health Check 2025. Available online at: https://www.planetaryhealthcheck.org/

[12] Planetary Health Alliance. (n.d.). What is planetary health? Available online at: https://planetaryhealthalliance.org/what-is-planetary-health/ (Accessed October 22, 2025).

[13] Redvers NF Guzman CA Parkes MW. Towards an educational praxis for planetary health: a call for transformative, inclusive, and integrative approaches for learning and relearning in the Anthropocene. Lancet Planetary Health. (2023) 7:E77–85. doi: 10.1016/S2542-5196(22)00332-1, 36608953

[14] Interprofessional Education Collaborative [IPEC]. (n.d.). What is interprofessional education? Available online at: https://www.ipecollaborative.org/about-us (Accessed October 22, 2025)

[15] Purdue University. (n.d.). What is transdisciplinarity? Available online at: https://www.purdue.edu/provost/innovation-hub/what-we-do/why-transdisciplinary/ (Accessed October 22, 2025)

[16] American Nurses Association. (2025). Code of ethics for nurses. Silver Spring, MD USA: American Nurses Association. Available online at: https://codeofethics.ana.org/home (accessed October 22, 2025).

[17] World Health Organization. (1978). Declaration of Alma Ata. Available online at: https://www.who.int/publications/i/item/WHO-EURO-1978-3938-43697-61471 (Accessed October 22, 2025).

[18] Planetary Health Alliance. (n.d.). Planetary health education. Available online at: https://planetaryhealthalliance.org/education-overview/ (Accessed October 22, 2025).

[19] Faerron Guzman C. Potter T. Planetary Health Alliance. (2021). Planetary health education framework. Available online at: https://planetaryhealthalliance.org/wp-content/uploads/2025/02/The-Planetary-Health-Education-Framework.pdf (accessed October 22, 2025).

[20] Planetary Health Alliance. (2021). Sao Paulo declaration on planetary health. Available online at: https://planetaryhealthalliance.org/wp-content/uploads/2025/02/Sao-Paulo-Declaration-on-Planetary-Health-Updated.pdf (accessed October 22, 2025).

[21] Planetary Health Alliance. (2024). Planetary health roadmap and action plan. Available online at: https://planetaryhealthalliance.org/wp-content/uploads/2025/02/Roadmap-to-Planetary-Health-FINAL.pdf (accessed October 22, 2025).

[22] Potter T. Burton E. Astle B. Honegger Rogers H. (2025). Nursing for planetary health and well-being. International Council of Nurses. Available online at: https://www.icn.ch/sites/default/files/2025-05/Planetary%20Health%20Topic%20Brief%20-%20EN_0.pdf (accessed October 22, 2025).

[23] International Council of Nurses. (2025). Nursing Student Steering Group and ICN Alliance of Student and Early Career Nurses. Planetary health and climate change. Available online at: https://www.icn.ch/sites/default/files/2025-05/Planetary%20Health%20SECN%20statement%20-%20EN_0.pdf (accessed October 22, 2025).

[24] Planetary Health Report Card. (n.d.). About us. Available online at: https://phreportcard.org/about/ (Accessed October 22, 2025)

[25] Planetary Health Report Card. (2025). Planetary health report card: 2024–2025 international summary report. Available online at: https://phreportcard.org/wp-content/uploads/2025/07/The-2025-Planetary-Health-Report-Card-International-Summary-Report-V5.pdf (Accessed December 10, 2025).

[26] Planetary Health Alliance. (n.d.). Teach planetary health. Available online at: https://planetaryhealthalliance.org/for-educators/ (Accessed December 10, 2025).

[27] Richardson K Steffen W Lucht W Bendtsen J Cornell SE Donges JF . Earth beyond six of nine Planetary Boundaries. Science Advances. (2023) 9:1–16. 10.1126/sciadv.adh2458 PMC10499318 37703365

[28] Rockström J Steffen W Noone K Persson A Chapin FS Lambin EF . A safe operating space for humanity. Nature. (2009) 461:472–475. doi: 10.1038/461472a 19779433

[29] Steffen F Richardson K Rockström J Cornell SE Fetzer I Bennet EM . Planetary boundaries: Guiding human development on a changing planet. Scienc. (2015) 341:1–10. 10.1126/science.1259855 25592418

